# Novel Identification of *Dermacentor variabilis* Arp2/3 Complex and Its Role in Rickettsial Infection of the Arthropod Vector

**DOI:** 10.1371/journal.pone.0093768

**Published:** 2014-04-14

**Authors:** Natthida Petchampai, Piyanate Sunyakumthorn, Mark L. Guillotte, Victoria I. Verhoeve, Kaikhushroo H. Banajee, Michael T. Kearney, Kevin R. Macaluso

**Affiliations:** Vector-borne Disease Laboratories, Department of Pathobiological Sciences, School of Veterinary Medicine, Louisiana State University, Baton Rouge, Louisiana, United States of America; Metabiota, United States of America

## Abstract

Tick-borne spotted fever group (SFG) *Rickettsia* species must be able to infect both vertebrate and arthropod host cells. The host actin-related protein 2/3 (Arp2/3) complex is important in the invasion process and actin-based motility for several intracellular bacteria, including SFG *Rickettsia* in *Drosophila* and mammalian cells. To investigate the role of the tick Arp2/3 complex in tick-*Rickettsia* interactions, open reading frames of all subunits of the protein including Arp2, Arp3, ARPC1, ARPC2, ARPC3, ARPC4, and ARPC5 were identified from *Dermacentor variabilis*. Amino acid sequence analysis showed variation (ranging from 25–88%) in percent identity compared to the corresponding subunits of the complex from *Drosophila melanogaster*, *Mus musculus*, *Homo sapiens*, and *Saccharomyces cerevisiae*. Potential ATP binding sites were identified in *D. variabilis* (*Dv*) Arp2 and Arp3 subunits as well as five putative WD (Trp-Asp) motifs which were observed in *Dv*ARPC1. Transcriptional profiles of all subunits of the *Dv*Arp2/3 complex revealed greater mRNA expression in both *Rickettsia*-infected and -uninfected ovary compared to midgut and salivary glands. In response to *R. montanensis* infection of the tick ovary, the mRNA level of only *Dv*ARPC4 was significantly upregulated compared to uninfected tissues. Arp2/3 complex inhibition bioassays resulted in a decrease in the ability of *R. montanensis* to invade tick tissues with a significant difference in the tick ovary, indicating a role for the Arp2/3 complex in rickettsial invasion of tick cells. Characterization of tick-derived molecules associated with rickettsial infection is imperative in order to better comprehend the ecology of tick-borne rickettsial diseases.

## Introduction

Ticks serve as both the transmission vectors and reservoir hosts for members of the obligate intracellular spotted fever group (SFG) *Rickettsia*. In tick populations, horizontal transmission of rickettsiae between ticks can occur via vertebrate hosts; vertical transmission between life cycle stages and transovarial transmission facilitates maintenance of the infection in tick hosts. Some tick species such as the American dog tick, *Dermacentor variabilis*, are associated with horizontal transmission of pathogenic SFG *Rickettsia* (*e.g. Rickettsia rickettsii*) [1], as well as vertical transmission of organisms with limited or no pathogenicity to humans (*e.g. Rickettsia montanensis*) [2]–[6]. The route of transmission correlates to the pathogenicity of the SFG *Rickettsia* and the impact on tick fitness, yet the influence of constitutive and introduced rickettsial infections in tick populations is understudied.

Towards understanding the process of SFG *Rickettsia* infection of ticks, several studies have surveyed the tick biological or immune response to infection [7]–[12]; however, at the molecular level little is known about the process of tick cell infection by SFG *Rickettsia*. It is recognized that rickettsiae enter host cells through receptor-mediated endocytosis [13]–[14]. Tick-derived histone H2B was demonstrated to play a role in tick cell infection by a non-SFG species, *R. felis*, in a tick-derived cell line [15], corroborating findings of a role for nuclear proteins in SFG *Rickettsia* mammalian cell invasion [16]. More recently, dysregulation of tick-derived α-catenin [17] and vacuolar-ATPase [18] were associated with rickettsial infection of tick-derived cell lines and whole organs. The host-derived molecules critical to cell infection by SFG *Rickettsia* have been examined in mammalian and *Drosophila* cells [16], [19]–[22]. Despite differences between host molecules associated with rickettsial entry in vertebrate and invertebrate hosts, the actin-related protein 2/3 (Arp2/3) complex is recognized as a central molecule stimulated during the internalization of SFG *Rickettsia* into host cells, independent of cell origin.

As a multi-subunit protein complex, Arp2/3 is composed of Arp2, Arp3, ARPC1, ARPC2, ARPC3, ARPC4 and ARPC5 [23]–[24]. The complex nucleates a new actin filament from the site of an existing filament. Supported by ARPC1–5, Arp2 and Arp3 are actin-related proteins that undergo conformational change and bind ATP. Arp2 and Arp3, combined with ATP hydrolysis, are required for Arp2/3 complex-mediated actin cytoskeleton remodeling [25]–[30]. In vertebrate and some insect cell lines, the Arp2/3 complex is a multi-functional protein essential for the invasion process of several pathogens such as *Listeria monocytogenes* [31]–[32], *Candida albicans*, *Escherichia coli* [33], *Chlamydia trachomatis* [34]–[36], *Yersinia pseudotuberculosis* [37], *Salmonella enterica* Typhimurium [38], *Pseudomonas aeruginosa* [39], and SFG *Rickettsia* [16], [21]. The complex is also shown to be important in actin-based motility of intracellular pathogens such as *L. monocytogenes* and *Shigella flexneri* [40]. While the evidence from vertebrate and insect cell culture models suggests an association between SFG *Rickettsia* and host Arp2/3, the presence of a tick Arp2/3 complex and its role in SFG *Rickettsia* infection of arthropod vectors remains undefined.

The recognized central role for Arp2/3 complex in invasion for several bacterial pathogens compelled our examination of the molecular characteristics of the tick Arp2/3 complex to determine the role of the protein in SFG *Rickettsia* invasion of the natural tick host. Novel gene sequences for all seven subunits of the Arp2/3 complex from *D. variabilis* were isolated and compared to other species. Also, transcriptional profiles of the Arp2/3 complex subunits in unexposed and *R. montanensis*-exposed tick tissues (midgut, ovary, and salivary glands) were investigated. Additionally, to test the hypothesis that the Arp2/3 complex is important in rickettsial invasion of tick cells, biochemical inhibition assays were conducted *ex vivo*. The functional study of the tick Arp2/3 complex at the tissue level provides insight into the molecular mechanisms of SFG *Rickettsia* infection in natural vector hosts.

## Materials and Methods

### Ethics Statement

The animal care and use performed during the following experiments was approved by the Louisiana State University Institutional Animal Care and Use Committee (Protocol Number: 10-035).

### Ticks and Tissue Recovery

*Rickettsia*-free *D. variabilis* colonies were maintained on vertebrate hosts at Louisiana State University, School of Veterinary Medicine as previously described [41]. For all bioassays, unfed or partially-fed (4 days) unmated female ticks were washed with 1% bleach (5 min), 70% ethanol (2 min), and 1% benzalkonium chloride (5 min). The ticks were rinsed once with sterile water between each wash and rinsed three times after the final wash. After air-drying, tick midgut, ovary, and salivary glands were excised and washed in sterile phosphate buffered saline (PBS, pH 7.4). For RNA extraction, buffer RLT (QIAGEN, Germantown, MD) or TRIzol reagent (Invitrogen, Carlsbad, CA) was added; tissues were passed through 27G needles or homogenized by grinding with plastic pestles for several minutes. The lysates were immediately used or stored at −80°C. For invasion assays, each tissue was placed individually into 1.7 ml microcentrifuge tubes containing 200 μl of L15C medium supplemented with 10% fetal bovine serum (Hyclone, Waltham, MA), 5% tryptose phosphate broth (Difco, Sparks, MD), 0.1% lipoprotein-cholesterol concentrate (LPC, MP Biomedicals, Santa Ana, CA), 0.6% HEPES solution (1 M, Sigma, St. Louis, MO), and 1.2% sodium bicarbonate solution (5%, Sigma). The samples were kept on ice until used in bioassays on the same day.

### *Rickettsia* Propagation and Tick Infection Procedures

*Rickettsia rickettsii* isolate Sheila Smith [42] and *R. montanensis* isolate M5/6 [43] were propagated in an African green monkey kidney cell line (Vero E6) cells cultured in Dulbecco’s modified Eagle’s medium (DMEM) high glucose (Invitrogen) containing 5% fetal bovine serum (Hyclone) and maintained in a humidified 5% CO_2_ incubator at 34°C. To generate a cDNA library, ticks were infected with *R. rickettsii* by needle inoculation. Briefly, frozen stock of *R. rickettsii* infected Vero cells (∼95% of the monolayer was infected) were thawed and centrifuged at 16000×g for 10 min. The cell pellet was reconstituted in 500 μl PBS and an equal aliquot was used to inject five unfed female ticks at the area between Coxa I and basis capituli. The injected ticks were kept at room temperature for 1 h prior to tissue removal. For organ specific invasion assays, *R. montanensis* was semi-purified from host cells using a modified protocol of Weiss et al. [44] as previously described [18]. The number of rickettsiae was enumerated by counting *Rickettsia* stained with a LIVE/DEAD BacLight Bacterial Viability Kit (Molecular Probes, Carlsbad, CA) in a Petroff–Hausser bacterial counting chamber (Hausser Scientific, Horsham, PA) and examined with a Leica microscope (Buffalo Grove, IL) [45].

### Cloning of the Tick Arp2/3 Complex Subunit Full-length cDNAs

The full-length cDNA for all seven subunits of Arp2/3 complex were identified in cDNA libraries generated from unfed (*R. rickettsii*-infected) or partially-fed (uninfected) *D. variabilis* using the SMARTer RACE cDNA Amplification Kit (Clontech, Mountain View, CA) or the GeneRacer Kit (Invitrogen), respectively, according to the manufacturers’ instructions. RACE-ready cDNAs were synthesized from total or mRNA using iScript reverse transcription kit (Bio-Rad, Hercules, CA) or SuperScript III Reverse Transcriptase (Invitrogen). Both 5′- and 3′- end fragments of the Arp2/3 complex subunits were amplified utilizing primers as shown in Table S1. Amplicons were cloned into pCR4-TOPO vector and transformed into TOP10 *E. coli* (Invitrogen). The plasmids were isolated and sequenced at Louisiana State University, School of Veterinary Medicine. Sequence of DNA was analyzed using BioEdit software and similarity comparison was carried out against protein database in GenBank using BlastX. Amino acid sequence analyses were conducted using web-based software suits. Multiple sequence comparison by log-expectation (MUSCLE, http://www.ebi.ac.uk/Tools/msa/muscle/) was used to create sequence alignment files and to calculate the percent identity matrix (created by Clustal2.1). The alignment output was created using GeneDoc software. ATP binding sites were predicted using NsitePred web server [46] and the conserved regions in proteins were identified by using the Simple Modular Architecture Research Tool (SMART, http://smart.embl-heidelberg.de/).

### Transcriptional Analysis during *Rickettsia* Infection

To determine the transcriptional profiles of the Arp2/3 complex subunit genes (all subunits) in dissected *D. variabilis* tissues from unfed females during *Rickettsia* infection, tick tissues (midgut, ovary, and salivary glands) were excised and exposed to *R. montanensis* (8×10^7^ per tissue) or complete L15C medium (uninfected groups). The samples were centrifuged at 4°C, 700×g for 2 min to facilitate the binding between *Rickettsia* and tick tissues. Rickettsiae were allowed to infect the tissues at 32°C for 1 h. The samples were then washed twice with 1 ml PBS and collected by centrifugation at 4°C, 275×g for 4 min. While using dissecting microscope, the supernatant was removed, leaving each tissue in each tube. Three samples of the same tissues were pooled and placed in 800 μl TRIzol reagent for RNA and DNA extraction as described in the manufacturer’s protocol. First-strand cDNA was then synthesized from 75 ng of DNase-treated total RNA using iScript reverse transcription kit (Bio-Rad) according to manufacturer’s instruction. Quantitative PCRs (qPCRs) were then performed using gene-specific primers (Table S2) for each subunit of the *Dv*Arp2/3 complex and the housekeeping gene, glyceraldehyde-3-phosphate dehydrogenase (GAPDH). All qPCR reactions were prepared in 96-well plates in a 35 μl volume composed of 0.1 μM each forward and reverse primers, DNase/RNase-free water, 2 μl of cDNA (sample) or water (negative control) and 2X LightCycler 480 SYBR Green I Master (Roche, Indianapolis, IN). The mixtures were aliquoted in triplicate 10 μl reactions onto 384-well plates and run on LightCycler 480 system II (Roche). Quantitative PCR assay conditions consisted of a 95°C pre-incubation for 10 min, 35 amplification cycles of 95°C for 15 sec, 60°C for 30 sec, and 72°C for 5 sec followed by a melting curve step of 95°C for 5 sec and 65°C for 1 min. A no RT reaction (water was added instead of reverse transcriptase) was performed to confirm an absence of genomic DNA (gDNA). Analyses of the crossing point (Cp) ratio of target (*Dv*Arp2, *Dv*Arp3, *Dv*ARPC1, *Dv*ARPC2, *Dv*ARPC3, *Dv*ARPC4, and *Dv*ARPC5) and reference (GAPDH) gene values were conducted with LightCycler 480 (1.5.0) software (Roche) using Basic Relative Quantification analysis (ΔΔCT-Method; Roche). Data are presented as the ratio of a target cDNA sequence to a reference cDNA sequence. To confirm the infection of tissues in the assays, DNA was extracted from the same samples after RNA isolation. Copies of rickettsial outer membrane protein B gene (*Rm*OmpB) were quantified using qPCR as previously described [18]. The infection experiments were performed twice independently.

### Arp2/3 Complex Inhibition Assays

A whole organ infection bioassay was developed based on a modified protocol of Bell [47]. Briefly, tick tissues including midgut, ovary, and salivary glands, placed individually in 1.7 ml microcentrifuge tubes, were treated with 500 μM CK-666 (EMD Millipore, Billerica, MA), an Arp2/3 complex inhibitor that binds between Arp2 and Arp3 subunits to prevent the complex’s ability to nucleate actin, and incubated at 32°C. After 3 h, *R. montanensis* (8×10^7^ per tissue) was used to infect tick tissues for 1 h. To remove excess extracellular rickettsiae, the tissues were then washed twice with 1 ml PBS and collected by low-speed centrifugation as described above. Genomic DNA was then extracted from the samples using the DNeasy Blood & Tissue Kit (QIAGEN) and eluted with 35 μl DNase/RNase free water. The numbers of rickettsiae and tick cells were then quantified using probe-based qPCR as previously described [18]. The experiments were performed in quadruplicate for each treatment group and the results were the combination of the three independent experiments.

### Statistical Analysis

Analysis of Variance (ANOVA) was conducted using the SAS statistical package (Version 9.3) GLM procedure. For transcriptional analysis, relative gene expression was analyzed using a two-way interaction (rickettsial infection and tick tissues). Pairwise t tests of least-squares means were used to examine the interaction effects of relative mRNA expression of each subunit of the *Dv*Arp2/3 complex between *Rickettsia*-exposed and -unexposed tissues or between tissues (midgut, ovary, salivary gland). For biochemical inhibition assays, the same tests were used to study a role of *Dv*Arp2/3 complex during rickettsial invasion of tick tissues. *P*-values of ≤0.05 were considered significantly different.

## Results

### Cloning and Sequence Analysis of *Dv*Arp2/3 Complex Subunits

Full-length cDNA clones corresponding to the transcript of *Dv*Arp2/3 complex subunit genes (*Dv*Arp2, *Dv*Arp3, *Dv*ARPC1, *Dv*ARPC2, *Dv*ARPC3, *Dv*ARPC4, and *Dv*ARPC5) from *D. variabilis* were isolated. The GenBank accession numbers, open reading frame (ORF) lengths, number of deduced amino acid sequences, and estimated molecular weights (MW) of each of the *Dv*Arp2/3 complex subunits are shown in Table 1.

**Table 1:** GenBank accession numbers, ORF size, amino acid sequence lengths, and estimated MW of *Dv*Arp2/3 complex subunits.

Subunit	GenBank accession numbers	ORF (bp)	Numbers of amino acids	Estimated MW (kDa)
*Dv*Arp2	KF780484	1191	396	45
*Dv*Arp3	KF780485	1230	409	46
*Dv*Arpc1	KF780486	1134	377	42
*Dv*Arpc2	KF780487	903	300	35
*Dv*Arpc3	KF780488	546	181	20
*Dv*Arpc4	KF780489	507	168	20
*Dv*Arpc5	KF780490	459	152	17

**Figure 1:**
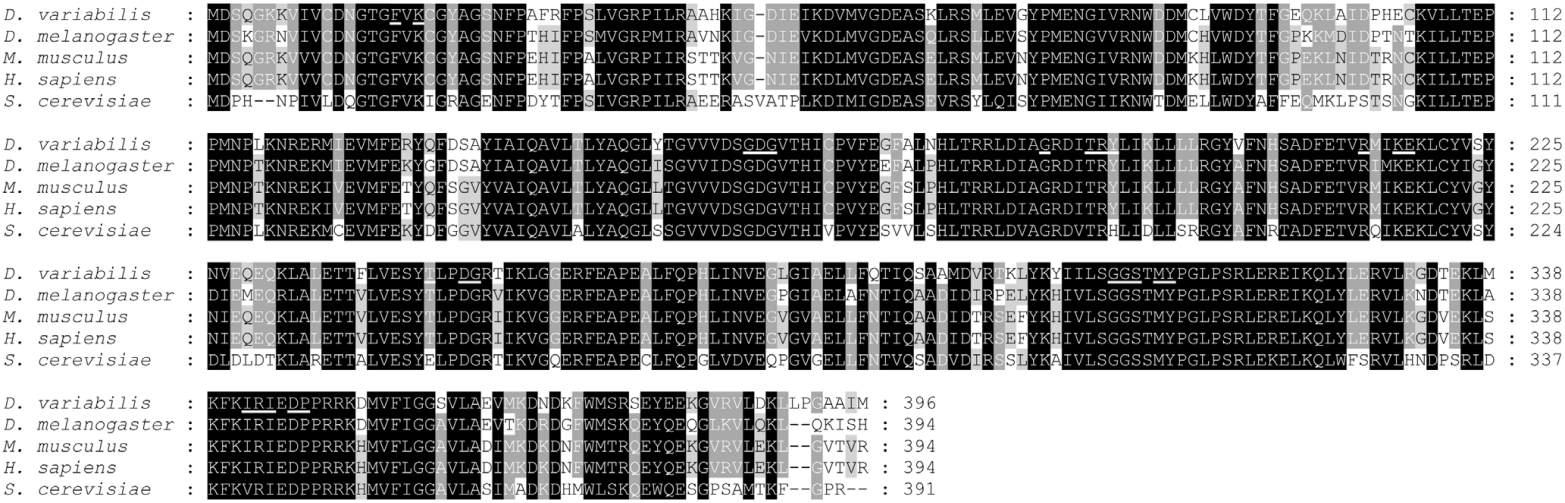
Tick Arp2 subunit multiple sequence alignment and identification of conserved ATP binding sites. Multiple sequence comparison by log-expectation (MUSCLE) software was used to create a sequence alignment of Arp2 subunits from *D. variabilis*, *D. melanogaster*, *M. musculus*, *H. sapiens*, and *S. cerevisiae*. Identical and similar amino acids are highlighted in black and grey, respectively. Conserved ATP binding sites predicted by the NsitePred web server are underlined.

**Figure 2:**
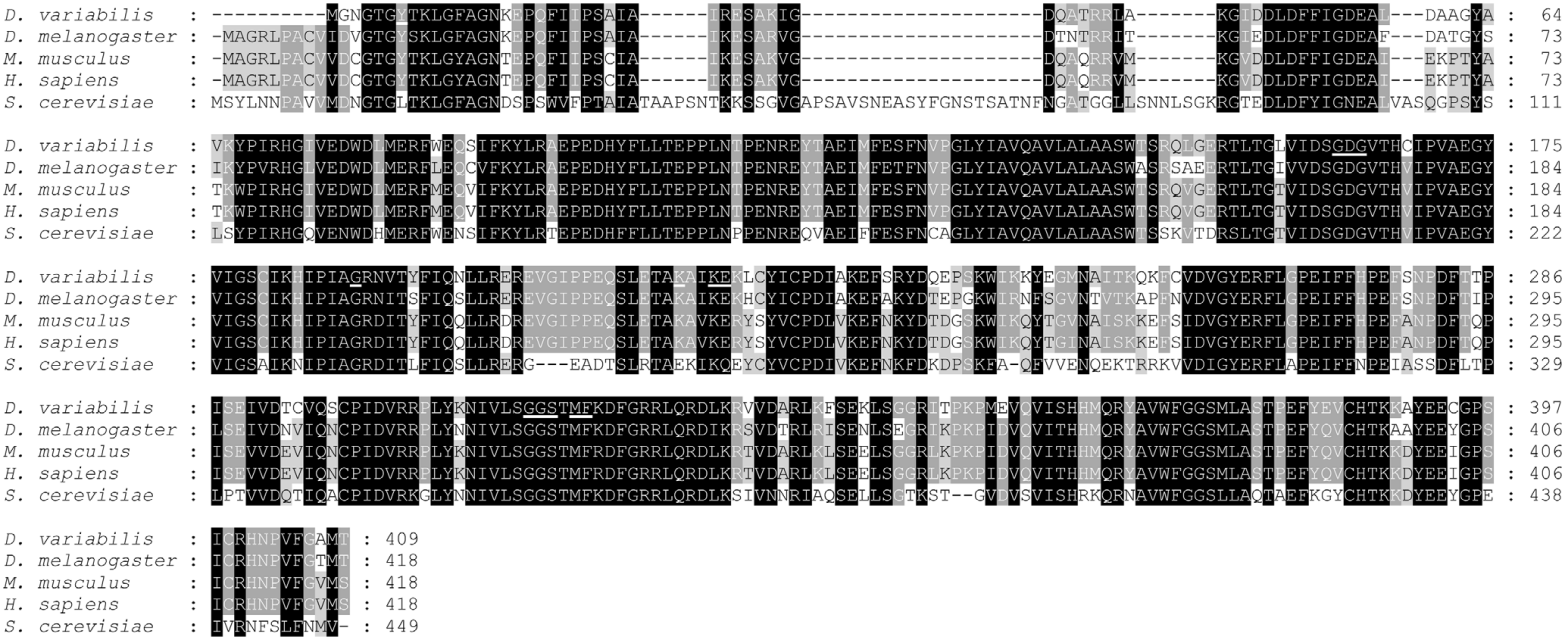
Tick Arp3 subunit multiple sequence alignment and identification of conserved ATP binding sites. Sequence alignment of Arp3 subunits from *D. variabilis*, *D. melanogaster*, *M. musculus*, *H. sapiens*, and *S. cerevisiae* was obtained from multiple sequence comparison by log-expectation (MUSCLE) software. Identical and similar amino acids are highlighted in black and grey, respectively. Conserved ATP binding sites predicted by NsitePred web server are underlined.

**Figure 3:**
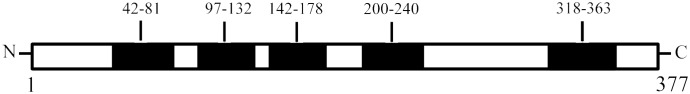
Schematic diagram represented the structure of *Dv*ARPC1 subunit with putative WD domains characteristic of ARPC1 subunit. Numbers correspond to amino acids of the protein sequence determined from the novel *D. variabilis* ARPC1 ORF. Shaded black regions are putative WD domains predicted by SMART software.

Amino acid sequence analyses of *Dv*Arp2/3 complex subunits were performed using a web-based multiple sequence alignment (MUSCLE) and the percent identity compared to the corresponding subunits of the Arp2/3 complex from *Drosophila melanogaster*, *Mus musculus*, *Homo sapiens*, and *Saccharomyces cerevisiae* are shown in Table 2. For each subunit the similarity ranged from 25–88%. Because Arp2 and Arp3 bind to ATP, the proteins were analyzed for ATP binding sites using NsitePred web server. Putative ATP-binding sites were identified for both Arp2 (Figure 1, underlined) and Arp3 (Figure 2, underlined) molecules, suggesting conserved activity among homologs. As shown in Figure 3, five putative WD (Trp-Asp) motifs which are conserved domains in ARPC1 protein [48], were also identified in the ARPC1 subunit from *D. variabilis*. Alignments for the remaining subunits, *Dv*ARPC1, *Dv*ARPC2, *Dv*ARPC3, *Dv*ARPC4, and *Dv*ARPC5 are provided in Figures S1–S5.

**Table 2:** Percent identity of *Dv*Arp2/3 complex subunits compared to the corresponding subunits of Arp2/3 complex from different organisms.

Subunit	*D. melanogaster* (%)	*M. musculus* (%)	*H. sapiens* (%)	*S. cerevisiae* (%)
***Dv*** **Arp2**	80	81	81	65
***Dv*** **Arp3**	83	83	83	64
***Dv*** **ARPC1**	56	56	56	40
***Dv*** **ARPC2**	79	78	78	40
***Dv*** **ARPC3**	68	66	66	47
***Dv*** **ARPC4**	83	88	88	66
***Dv*** **ARPC5**	60	56	56	25

### Expression of *Dv*Arp2/3 Complex Subunit mRNAs in Tick Tissues Infected *Ex vivo*

**Figure 4:**
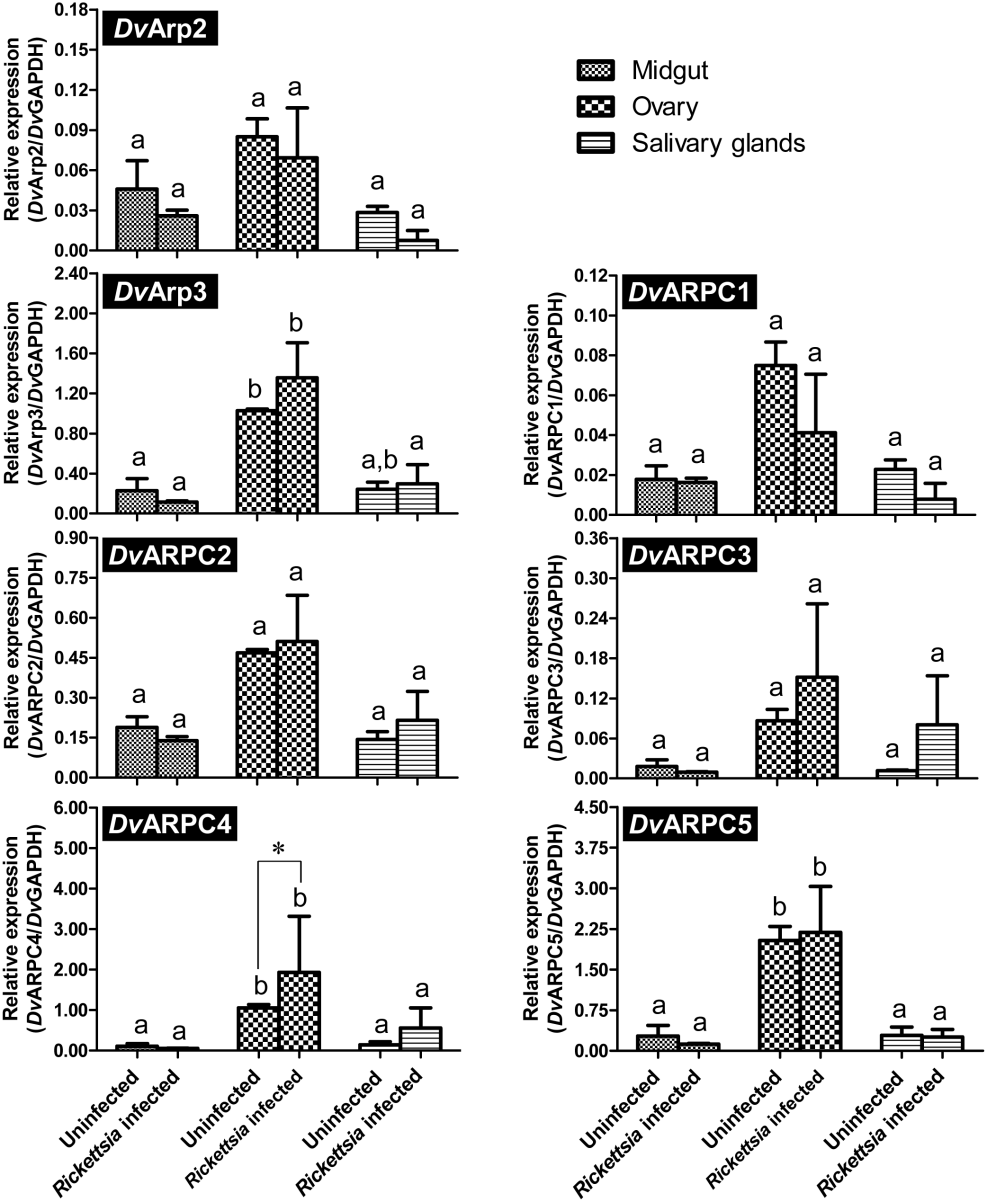
Transcriptional profile of Arp2/3 complex (all subunits) in *D. variabilis* tissues. *R. montanensis* was used to infect tick midgut, ovary, and salivary glands (8×10^7^ rickettsiae per tissue) for 1 h. After removal of rickettsiae, tick tissues were washed and collected by low-speed centrifugation. Total RNA was then extracted from the tissues and the levels of *Dv*Arp2/3 complex mRNA were measured by qRT-PCR. *Dv*GAPDH mRNA was used to normalize the differences among samples. Data shown are mean (±SEM) relative expression from two independent experiments. The asterisk denotes a significant difference between treatment groups (unexposed- or *Rickettsia-*exposed group) in the same tissue. For each subunit, different letters above bars represents significance differences between tissues.

To define the transcriptional profiles of the *Dv*Arp2/3 complex (all subunits) in *D. variabilis* tissues (midgut, ovary, and salivary glands) in response to *R. montanensis* infection, tick tissues were dissected out of the ticks and exposed to rickettsiae. Transcriptional activity of *Dv*Arp2, *Dv*Arp3, *Dv*ARPC1, *Dv*ARPC2, *Dv*ARPC3, *Dv*ARPC4, and *Dv*ARPC5 mRNA were measured by quantitative reverse-transcription (qRT)-PCR. The mRNA of all *Dv*Arp2/3 complex subunits was detectable in all tick tissues, and in both *R. montanensis*-exposed and -unexposed tissues (Figure 4). Interestingly, the mRNA levels were expressed at a greater level in the ovary compared to the midgut and salivary glands with significant differences for *Dv*Arp3 (*P* = 0.0496 in uninfected ovary compared to midgut; *P* = 0.0031 and 0.0105 in infected ovary compared to midgut and salivary glands, respectively), *Dv*ARPC4 (*P* = 0.0217 and 0.0270 in uninfected ovary compared to midgut and salivary glands, respectively; *P*<0.0001 and *P* = 0.0012 in infected ovary compared to midgut and salivary glands, respectively), and *Dv*ARPC5 (*P*<0.0001 in uninfected ovary compared to both midgut and salivary glands; *P*<0.0001 in infected ovary compared to both midgut and salivary glands). The transcription of *Dv*ARPC4 was significantly (*P* = 0.0311) upregulated in response to *R. montanensis* infection in the ovary, compared to uninfected tissues.

To confirm the infection of tick tissues in the assays, DNA was extracted from the same samples after RNA isolation and the copies of the rickettsial gene (*Rm*OmpB) in infected tissues were quantified by qPCR. The average numbers of invading *Rickettsia* from two independent experiments are 1.56×10^4^, 1.09×10^4^, and 1.93×10^4^, in midgut, ovary, and salivary glands, respectively.

### *Dv*Arp2/3 Complex Inhibition Assay

**Figure 5:**
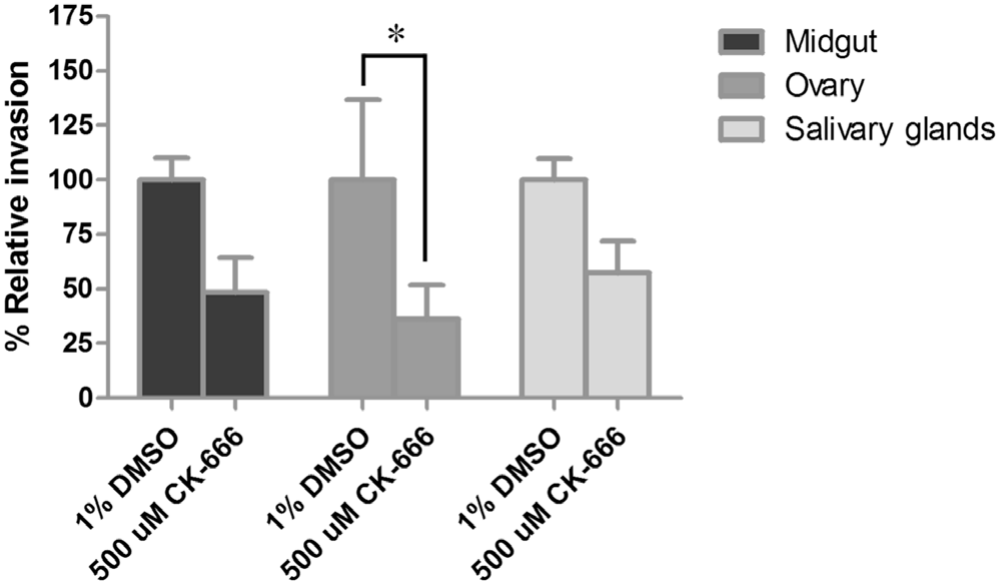
Effect of Arp2/3 complex inhibitor on *R. montanensis* invasion of *D. variabilis* tissues. Tick tissues including midgut, ovary, and salivary glands were dissected out prior to infection with *R. montanensis* (8×10^7^ per tissue). After 1 h, rickettsiae were removed and the tissues were washed once with PBS and rickettsiae and tick cells were quantified by qPCR. The experiments were performed in quadruplicate for each treatment group and the results were the combination of the three independent experiments. The asterisk indicates a significant difference between treatment and inhibitor vehicle control.

To further characterize the Arp2/3 complex in rickettsial infection of a vector host, an inhibition assay was performed in tick tissues. Midgut, ovary, and salivary glands were recovered from unfed female ticks and treated with 500 μM CK-666, an Arp2/3 complex inhibitor, for 3 h. *R. montanensis* was then used to infect the tissues (8×10^7^ per tissue) for 1 h, and the tissues were washed twice with PBS to remove extracellular rickettsiae. Genomic DNA was then extracted from the samples and the number of invading rickettsiae and tick cells were quantified by qPCR. Compared to inhibitor vehicle alone, the presence of CK-666 influenced rickettsial invasion by decreasing the number of rickettsiae entering the cells by as much as 70%. As shown in Figure 5, inhibition of *Dv*Arp2/3 complex resulted in a decrease in *R. montanensis* invasion of all tissues with significant difference (*P* = 0.0477) in the ovary.

## Discussion

The Arp2/3 complex is a seven-subunit protein actin nucleator widely expressed in eukaryotic cells. In order to invade host cells, several bacterial pathogens, including SFG *Rickettsia* [16], [21], [49]–[50], exploit the host Arp2/3 complex. For these tick-borne bacteria, this interactive process has been examined primarily *in vitro* with model systems without assessing the utility of the Arp2/3 complex in SFG *Rickettsia* infection in ticks. The current study provides the first molecular description of host machinery and utilization by rickettsiae in a competent vector host.

The current study provides the molecular and functional characterization of the Arp2/3 complex from *D. variabilis*, a competent vector of SFG *Rickettsia*. Full-length cDNAs encoding all seven subunits of the protein (*Dv*Arp2, *Dv*Arp3, *Dv*ARPC1, *Dv*ARPC2, *Dv*ARPC3, *Dv*ARPC4, and *Dv*ARPC5) were isolated, and multiple sequence alignments showed variation in percent identity compared to the corresponding subunits of the complex from *D. melanogaster*, *M. musculus*, *H. sapiens*, and *S. cerevisiae*. Although *Dv*ARPC1 is one of the more divergent subunits, conserved putative WD domains of ARPC1 [48] were observed in ARPC1 isolated from *D. variabilis*. The WD repeat, also known as the Trp-Asp or WD40 motif, is involved in a wide variety of cellular processes such as RNA processing, signal transduction, cytoskeleton assembly, and macromolecular protein complex formation [51]–[52]. Welch and colleagues [48] suggested the ARPC1 subunit influences assembly and maintenance of the Arp2/3 complex structure correlating with the capability of WD motif containing proteins in the coordination of multiprotein complexes. It was also postulated that ARPC1 facilitated the binding of the Arp2/3 complex with proteins that regulate its functions [48]. Furthermore, amino acid sequence analysis of *Dv*Arp2 and *Dv*Arp3 revealed putative ATP binding sites consistent with studies demonstrating that ATP binding on Arp2 and Arp3, as well as ATP hydrolysis on Arp2, were required for Arp2/3 complex-mediated actin cytoskeleton rearrangement [25]–[29]. Identification of the Arp2/3 complex subunits and the conserved nature of active subunits suggests ticks have a viable Arp2/3 complex.

Toward functional characterization in ticks, transcriptional profiles of *Dv*Arp2/3 complex subunits were examined in both *Rickettsia*-infected and -uninfected tick tissues. The results indicate mRNAs of all subunits are expressed at greater levels in the tick ovary (both in *Rickettsia*-infected and -uninfected ovary) than in midgut and salivary glands with significant difference for *Dv*Arp3 (in uninfected ovary compared to midgut only and in infected ovary compared to both midgut and salivary glands), *Dv*ARPC4, and *Dv*ARPC5. The abundant expression of *Dv*Arp2/3 complex transcripts implies an important role of this molecule in the tick ovary. In *Drosophila*, the Arp2/3 complex is essential for oogenesis; Hudson and Cooley [53] demonstrated *arp3* and *arpc1* mutants inhibit germ line nurse cells from transporting the cytoplasmic contents to the oocytes. The increased activity in the tick ovary is intriguing as SFG *Rickettsia* are vertically maintained in tick populations via transovarial transmission. If infection of the ovary results in increased Arp2/3 complex activity and, similar to *Drosophila*, Arp2/3 activity is associated with successful oogenesis, then a possible beneficial fitness effect might be associated with rickettsial infection in the tick system. The role of the Arp2/3 complex in the tick ovary relative to oogenesis requires thorough examination.

The Arp2/3 complex is important in the regulation of actin polymerization, a key process exploited by SFG *Rickettsia* to invade host cells, including *Drosophila* and mammalian cells [16], [21], [49]–[50]. During rickettsial invasion, the host cell Arp2/3 complex is activated by RickA, a nucleation-promoting factor mimic expressed by *Rickettsia* [50], [54]. Recently, transformed *Rickettsia bellii* that overexpressed *Rickettsia monacensis rickA* were internalized quicker than negative control transformants [55] suggesting a role for the Arp2/3 complex in rickettsial entry. Of the seven subunits of the Arp2/3 complex, studies have demonstrated that ARPC4 is the most crucial subunit for the complex assembly in vertebrates and *S. cerevisiae* [56]–[57]. In the current study, the mRNA level of *Dv*ARPC4 was shown to be significantly upregulated in response to the early stage of *R. montanensis* infection of the tick ovary implying the importance of this subunit during host cell invasion. However functions of the individual Arp2/3 complex subunits need to be confirmed at protein level. Because tick tissues including midgut, ovary, and salivary glands are essential for both horizontal and vertical transmission of SFG *Rickettsia* [58], the role of the *Dv*Arp2/3 complex was further studied at the protein level during *R. montanensis* infection of *D. variabilis*. Using an *ex vivo* bioassay, a decrease in percent relative rickettsial invasion was observed in all tick tissues treated with CK-666, a specific chemical inhibitor of the Arp2/3 complex [59]. When compared to untreated, control tissues, a significant decrease was realized in the tick ovary. The lack of complete abolition of invasion was not observed in CK-666-treated cells likely due to multiple factors including the inability for the inhibitor to reach every cell in the organ explants or, possibly, the rickettsiae use an alternate mechanism for infection. Compared to other studies using CK-666, inhibition of rickettsial infection of host cells is typically not 100% [21]. Thus, both transcriptional dysregulation and protein function suggest an essential role for the Arp2/3 complex during rickettsial invasion of tick tissues.

As a multifunctional protein, the Arp2/3 complex is also found to be important in actin-based motility of intracellular pathogens. For example, *L. monocytogenes* and *S. flexneri* express surface proteins that either mimic or activate host nucleation-promoting factors leading to the stimulation of the Arp2/3 complex and subsequent actin tail assembly and organization at the bacterial surface [40]. However, the importance of the complex in *Rickettsia* movement has been debated in the last decade [14], [50], [54]–[55], [60]–[64]. For example, *in vitro* studies utilizing *Rickettsia conorii* [50] and *R. rickettsii* [54] demonstrated that the activation of Arp2/3 complex by RickA facilitated actin nucleation and the organization of Y-branched actin networks. The roles for Arp2/3 complex in actin nucleation and Y-branched filament formation were proposed to be involved in an early stage of rickettsial movement [54]. In contrast, a knock-down of Arp2/3 complex subunits in a non-vector *Drosophila* cell model had only moderately impacted the length of *R. parkeri* actin tail formation, suggesting a non-essential role of the molecule in actin-based motility in *Drosophila* [64]. Further studies to investigate the role of the Arp2/3 complex in SFG *Rickettsia* movement in a vector host are required.

In summary, the present study provides the first description of all seven subunits of the tick-derived Arp2/3 complex and assigns a novel role for the protein in facilitating the uptake of *Rickettsia* into specific tick tissues. The current study also highlights several findings of importance; the mRNA level of the individual Arp2/3 complex subunits was expressed at a greater level in the ovary (both in *Rickettsia*-infected and -uninfected ovary) compared to the midgut and salivary glands. Likewise, *Dv*ARPC4 mRNA was significantly upregulated in response to rickettsial invasion of the tick ovary, and inhibition of the *Dv*Arp2/3 complex significantly decreased the entry of *Rickettsia* into the tick ovary. Further characterization of tick Arp2/3 complex is required for better understanding the precise mechanisms of the complex in rickettsial infection of arthropod vectors. Alternate inhibitions assays using CK-548, an Arp2/3 complex inhibitor specifically acting on the Arp3 subunit, or siRNA of individual subunits will allow a detailed analysis of the role and function of individual subunits of the Arp2/3 complex in the arthropod vector. Building upon the findings of the current study, the interaction between the Arp2/3 complex and SFG *Rickettsia* in regards to transmission by ticks requires further study.

## Supporting Information

Figure S1 **Multiple sequence alignment of ARPC1 subunit sequences.** Multiple sequence comparison by log-expectation (MUSCLE) software was utilized to generate sequence alignment of ARPC1 subunits from *D. variabilis*, *D. melanogaster*, *M. musculus*, *H. sapiens*, and *S. cerevisiae*. Identical and similar amino acids are highlighted in black and grey, respectively. The figure was created using GeneDoc software.
(TIF)

Figure S2 **Multiple sequence alignment of ARPC2 subunit sequences.** Sequence alignment of ARPC2 subunits from *D. variabilis*, *D. melanogaster*, *M. musculus*, *H. sapiens*, and *S. cerevisiae* was generated using multiple sequence comparison by log-expectation (MUSCLE) software. Identical and similar amino acids are highlighted in black and grey, respectively. The figure was created using GeneDoc software.
(TIF)

Figure S3 **Multiple sequence comparison of ARPC3 subunit.** The *Dv*ARPC3 deduced amino acid sequence was aligned *D. variabilis*, *D. melanogaster*, *M. musculus*, *H. sapiens*, and *S. cerevisiae*. Alignment was performed using multiple sequence comparison by log-expectation (MUSCLE) software. Shaded light red and dark red indicate identical and similar amino acid residues, respectively. The figure was created using GeneDoc software.
(TIF)

Figure S4 **Multiple sequence alignment of ARPC4 subunit sequences.** Sequence alignment of ARPC4 subunits from *D. variabilis*, *D. melanogaster*, *M. musculus*, *H. sapiens*, and *S. cerevisiae* was conducted using multiple sequence comparison by log-expectation (MUSCLE) software. Identical and similar amino acids are shaded in black and grey, respectively. The figure was created using GeneDoc software.
(TIF)

Figure S5 **Multiple sequence comparison of ARPC5 subunit of Arp2/3 complex.** Multiple sequence comparison by log-expectation (MUSCLE) software was used to produce sequence alignment of ARPC5 subunits from *D. variabilis*, *D. melanogaster*, *M. musculus*, *H. sapiens*, and *S. cerevisiae*. Identical and similar amino acids are highlighted in black and grey, respectively. The figure was created using GeneDoc software.
(TIF)

Table S1 **Primers used in full-length cDNA isolation of** ***Dv*** **Arp2/3 complex (all subunits).**
(DOCX)

Table S2 **Primers and probes used in qRT-PCR and qPCR assays.**
(DOCX)
