# Supplementary figures and images for: Novel Identification of Dermacentor variabilis Arp2/3 Complex and Its Role in Rickettsial Infection of the Arthropod Vector
Source: PLoS One. 2014 Apr 14;9(4):e93768. doi: 10.1371/journal.pone.0093768 (PMC3986078; doi:10.1371/journal.pone.0093768)

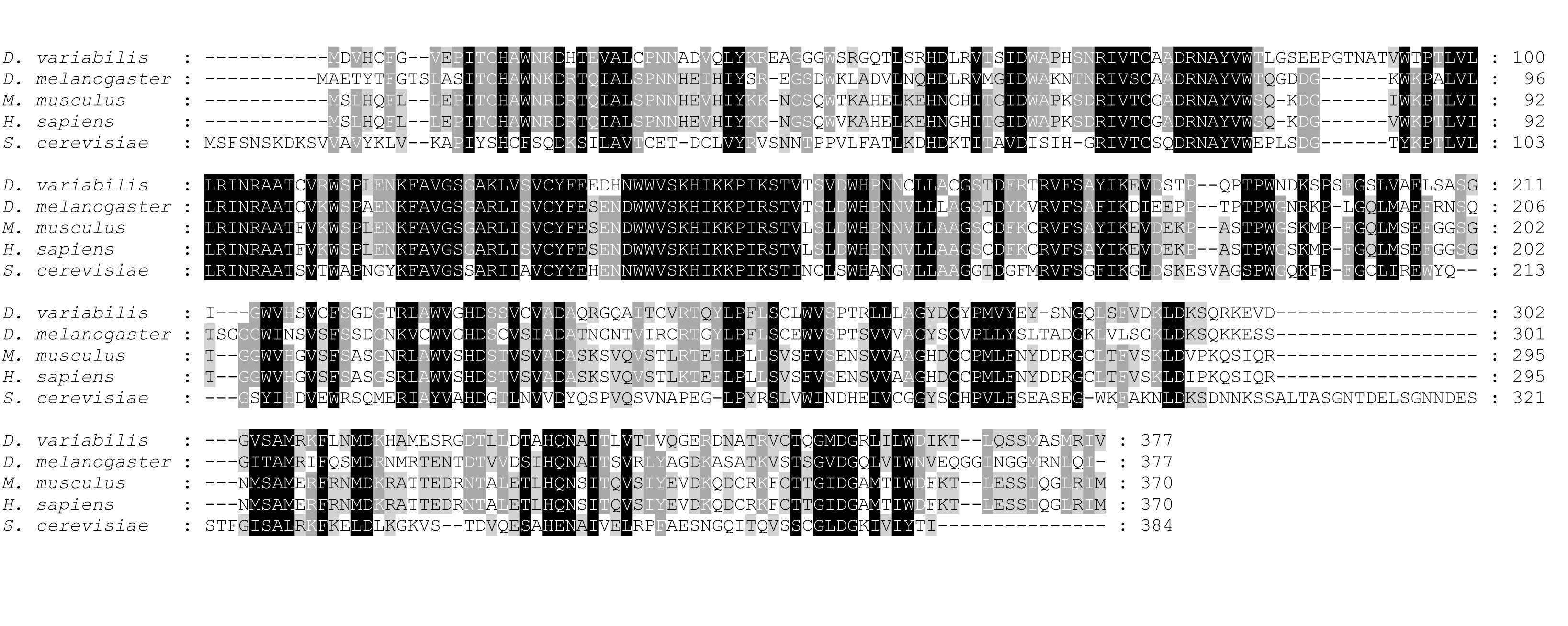

Supplement: Figure S1 — Multiple sequence alignment of ARPC1 subunit sequences. Multiple sequence comparison by log-expectation (MUSCLE) software was utilized to generate sequence alignment of ARPC1 subunits from D. variabilis, D. melanogaster, M. musculus, H. sapiens, and S. cerevisiae. Identical and similar amino acids are highlighted in black and grey, respectively. The figure was created using GeneDoc software. (TIF) [file pone.0093768.s001.tif]

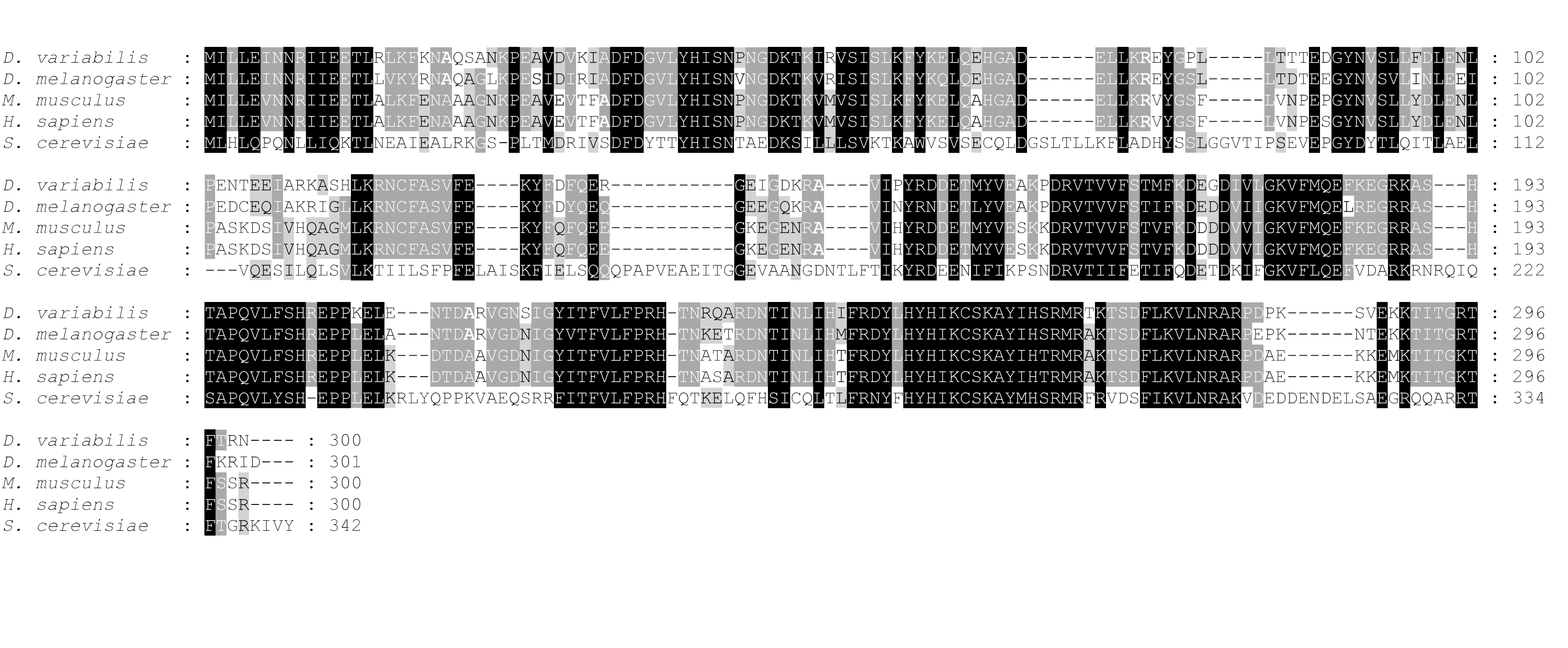

Supplement: Figure S2 — Multiple sequence alignment of ARPC2 subunit sequences. Sequence alignment of ARPC2 subunits from D. variabilis, D. melanogaster, M. musculus, H. sapiens, and S. cerevisiae was generated using multiple sequence comparison by log-expectation (MUSCLE) software. Identical and similar amino acids are highlighted in black and grey, respectively. The figure was created using GeneDoc software. (TIF) [file pone.0093768.s002.tif]

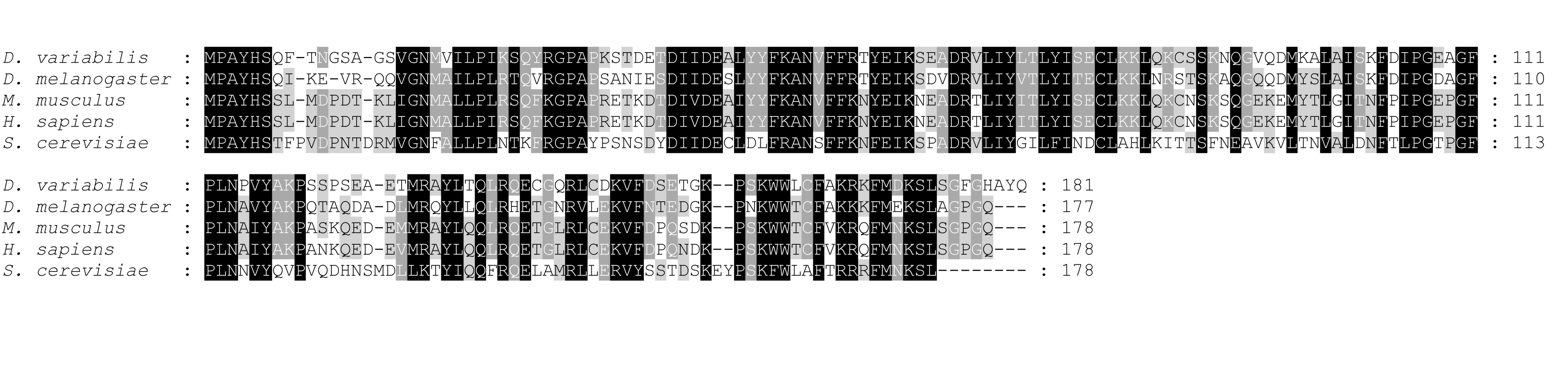

Supplement: Figure S3 — Multiple sequence comparison of ARPC3 subunit. The DvARPC3 deduced amino acid sequence was aligned D. variabilis, D. melanogaster, M. musculus, H. sapiens, and S. cerevisiae. Alignment was performed using multiple sequence comparison by log-expectation (MUSCLE) software. Shaded light red and dark red indicate identical and similar amino acid residues, respectively. The figure was created using GeneDoc software. (TIF) [file pone.0093768.s003.tif]

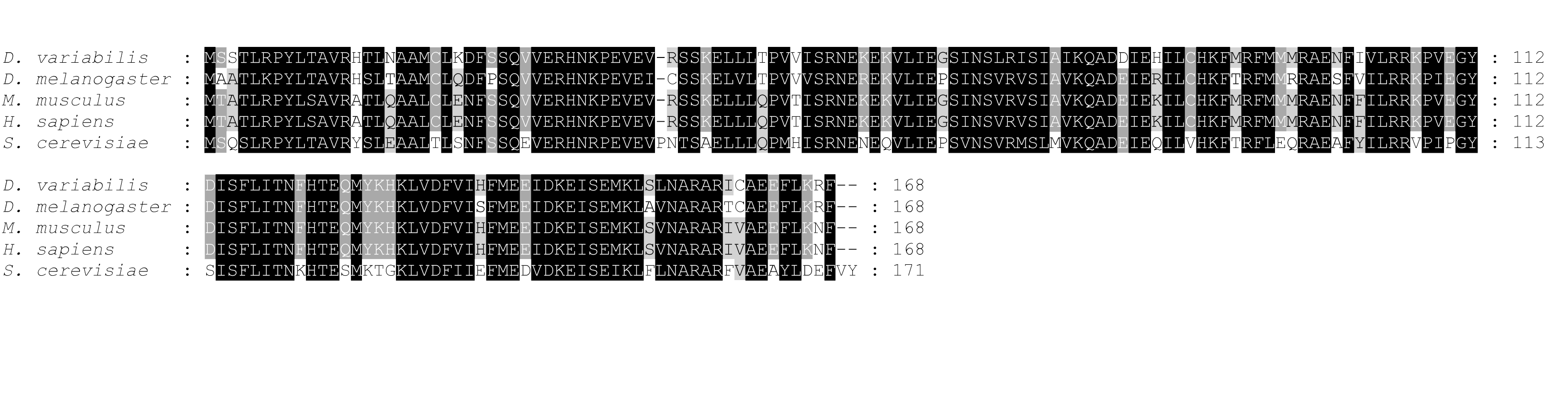

Supplement: Figure S4 — Multiple sequence alignment of ARPC4 subunit sequences. Sequence alignment of ARPC4 subunits from D. variabilis, D. melanogaster, M. musculus, H. sapiens, and S. cerevisiae was conducted using multiple sequence comparison by log-expectation (MUSCLE) software. Identical and similar amino acids are shaded in black and grey, respectively. The figure was created using GeneDoc software. (TIF) [file pone.0093768.s004.tif]

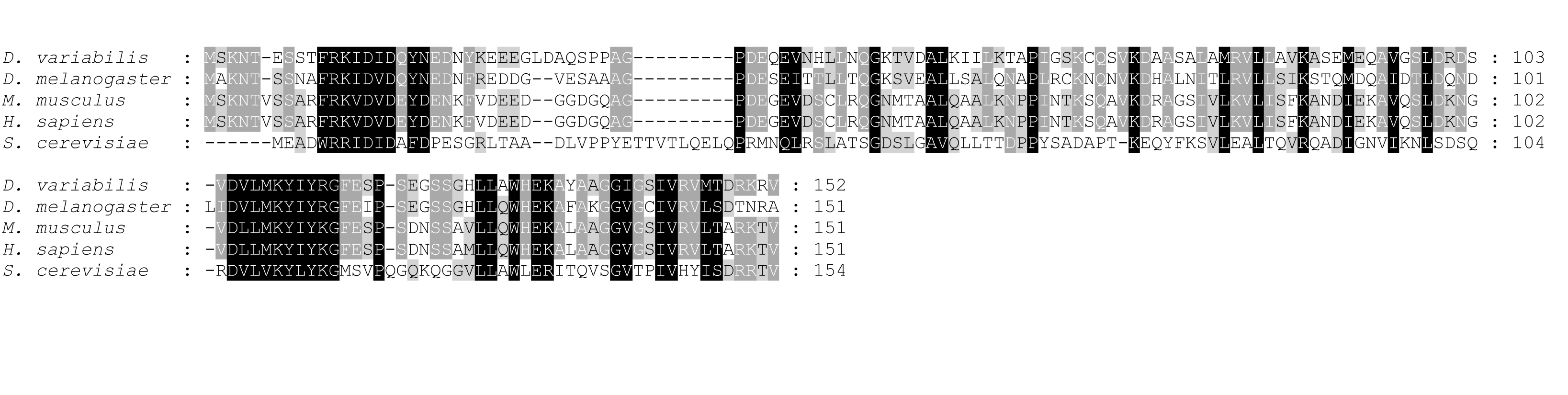

Supplement: Figure S5 — Multiple sequence comparison of ARPC5 subunit of Arp2/3 complex. Multiple sequence comparison by log-expectation (MUSCLE) software was used to produce sequence alignment of ARPC5 subunits from D. variabilis, D. melanogaster, M. musculus, H. sapiens, and S. cerevisiae. Identical and similar amino acids are highlighted in black and grey, respectively. The figure was created using GeneDoc software. (TIF) [file pone.0093768.s005.tif]
