# Supplementary material for: Novel Identification of Dermacentor variabilis Arp2/3 Complex and Its Role in Rickettsial Infection of the Arthropod Vector
Source: PLoS One. 2014 Apr 14;9(4):e93768. doi: 10.1371/journal.pone.0093768 (PMC3986078; doi:10.1371/journal.pone.0093768)
Supplement: Table S1 — Primers used in full-length cDNA isolation of Dv Arp2/3 complex (all subunits). (DOCX) [file pone.0093768.s006.docx]

**Table S1** Primers used in full-length cDNA isolation of *Dv*Arp2/3 complex (all subunits).

| Primers | Sequences (5’ to 3’) | Fragment obtained |
| --- | --- | --- |
| *Dv*Arp2 Fw | CCGCCGATGAACCCGCTCAAAAACC | 3’*Dv*Arp2 |
| *Dv*Arp2 Rev | CTCGATCATGCGCTCGCGGTTTTTG | 5’*Dv*Arp2 |
| *Dv*Arp3 Fw | GTCCCCCTGGGGCTGAGGGCTATGT | 3’DvArp3 |
| *Dv*Arp3 Rev | ATGCCAACATGCTGCCACCAAACCA | 5’*Dv*Arp3 |
| *Dv*ARPC1 Fw | TCACCTGCCACGCATGGAACAAGGA | 3’*Dv*ARPC1 |
| *Dv*ARPC1 Rev | CTTGGCACCTGACCCCACTGCAAAC | 5’*Dv*ARPC1 |
| *Dv*ARPC2 Fw | AACAAAGATAAGGGTCAGCA | 3’*Dv*ARPC2 |
| *Dv*ARPC2 Rev | TGCGCGCAATCTCCTCTGTGTTCT | 5’ *Dv*ARPC2 |
| *Dv*ARPC3 Fw | GTGGGCAACGTCTATGCGACAAGGT | 3’ *Dv*ARPC3 |
| *Dv*ARPC3 Rev | TTGTCGCATAGACGTTGCCCACACT | 5’*Dv*ARPC3 |
| *Dv*ARPC4 Fw | GATTCATGATGATGCGTGCCGAGAA | 3’*Dv*ARPC4 |
| *Dv*ARPC4 Rev | GCACAAATACGTGCCCTTGCATTGAG | 5’*Dv*ARPC4 |
| *Dv*ARPC5 Fw | GCTCCCATAGGCTCCAAATGCCAAA | 3’*Dv*ARPC5 |
| *Dv*ARPC5 Rev | GCTGGCTGCATCCTTTACACTTTGG | 5’*Dv*ARPC5 |
