# Supplementary material for: Novel Identification of Dermacentor variabilis Arp2/3 Complex and Its Role in Rickettsial Infection of the Arthropod Vector
Source: PLoS One. 2014 Apr 14;9(4):e93768. doi: 10.1371/journal.pone.0093768 (PMC3986078; doi:10.1371/journal.pone.0093768)
Supplement: Table S2 — Primers and probes used in qRT-PCR and qPCR assays. (DOCX) [file pone.0093768.s007.docx]

**Table S2** Primers and probes used in qRT-PCR and qPCR assays.

| Primers | Sequences (5’ to 3’) | Experiment | Reference |
| --- | --- | --- | --- |
| *Dv*Arp2_123_Fw  *Dv*Arp2_231_Rev | GGGGTTTGTCAAATGTGGTT  TTCGATGTCACCTATCTTGTGG | mRNA expression | This study |
| *Dv*Arp3_593_Fw  *Dv*Arp3_693_Rev | ATACGCTGTCTGGTTTGGTG  GATGCTCGGTCCACACTCTT | mRNA expression | This study |
| *Dv*Arpc1_226_Fw  *Dv*Arpc1_338_Rev | GCAGCTCTACAAGCGAGAGG  TCACAATGCGGTTACTGTGTG | mRNA expression | This study |
| *Dv*Arpc2_356_Fw  *Dv*Arpc2_460_Rev | AGGAACTGCTTTGCGTCTGT  CATGGTTTCATCGTCCCTGT | mRNA expression | This study |
| *Dv*Arpc3_477_Fw  *Dv*Arpc3_588_Rev | TTCCAGGAGAAGCTGGATTT  TGCCCACACTCT TGTCGTAG | mRNA expression | This study |
| *Dv*Arpc4_262_Fw  *Dv*Arpc4_474_Rev | GCAACGAGAAGGAGAAGGTG  TCTGCTCCGTATGGAAGT TTG | mRNA expression | This study |
| *Dv*Arpc5_375_Fw  *Dv*Arpc5_476_Rev | AGCCTTCCTCCTCCTTGTAGT T  CCGTTT CTCTGCTCA CTATGT CT | mRNA expression | This study |
| *Dv*GAPDH-926For  *Dv*GAPDH-1024Rev | ACTCCCACAGCAGCATCTTT  TGCTGTAGCCGTACTCGTTG | mRNA expression | This study |
| CRT*Dv*321F  CRT*Dv*452R  *Dv*CRT_TYE665 | AGGAGAAAAGCAAGGGACTG  CAATGTTCTGCTCGTGCTTG  TYE665/TGGAGAAGGGCTCGAACTTGGC/IAbRQSp | mRNA expression, inhibition assays | 18 |
| OmpB*Rm*2832F  OmpB*Rm*2937R  *Rm*OmpB_HEX | GCGGTGGTGTTCCTAATAC  CCTAAGTTGTTATAGTCTGTAGTG  HEX/CGGGGCAAAGATGCTAGCGCTTCACAGTTACCCCG/IABkFQ | mRNA expression, inhibition assays | 18 |
